# Interaction between Hydrogenase Maturation Factors HypA and HypB Is Required for [NiFe]-Hydrogenase Maturation

**DOI:** 10.1371/journal.pone.0032592

**Published:** 2012-02-27

**Authors:** Kwok-Ho Chan, Ka-Man Lee, Kam-Bo Wong

**Affiliations:** School of Life Sciences, Centre for Protein Science and Crystallography, The Chinese University of Hong Kong, Shatin, Hong Kong Special Administrative Region, People's Republic of China; Shantou University Medical College, China

## Abstract

The active site of [NiFe]-hydrogenase contains nickel and iron coordinated by cysteine residues, cyanide and carbon monoxide. Metal chaperone proteins HypA and HypB are required for the nickel insertion step of [NiFe]-hydrogenase maturation. How HypA and HypB work together to deliver nickel to the catalytic core remains elusive. Here we demonstrated that HypA and HypB from *Archaeoglobus fulgidus* form 1∶1 heterodimer in solution and HypA does not interact with HypB dimer preloaded with GMPPNP and Ni. Based on the crystal structure of *A. fulgidus* HypB, mutants were designed to map the HypA binding site on HypB. Our results showed that two conserved residues, Tyr-4 and Leu-6, of *A. fulgidus* HypB are required for the interaction with HypA. Consistent with this observation, we demonstrated that the corresponding residues, Leu-78 and Val-80, located at the N-terminus of the GTPase domain of *Escherichia coli* HypB were required for HypA/HypB interaction. We further showed that L78A and V80A mutants of HypB failed to reactivate hydrogenase in an *E. coli* Δ*hypB* strain. Our results suggest that the formation of the HypA/HypB complex is essential to the maturation process of hydrogenase. The HypA binding site is in proximity to the metal binding site of HypB, suggesting that the HypA/HypB interaction may facilitate nickel transfer between the two proteins.

## Introduction

Hydrogenase maturation factors HypA and HypB are involved in the delivery of nickel into the catalytic core of [NiFe]-hydrogenase [1], [2], [3], [4], [5], [6], [7]. Nickel and iron ions in the catalytic core of [NiFe]-hydrogenase are coordinated by two CN and one CO molecules and a network of cysteine residues [8]. Formation of the catalytic core of [NiFe]-hydrogenase requires accessory proteins encoded by genes designated *hypA* to *hypF* in the *hyp* operon. The Fe center is assembled before the insertion of nickel [9], [10], [11]. The hydrogenase maturation factors HypC, HypD, HypE and HypF are responsible for the synthesis and transfer of the Fe(CN)_2_ complex to the hydrogenase precursor [12], [13], [14], [15]. The mechanism of CO ligand synthesis is unclear [16], [17], [18]. After delivery of the Fe(CN)_2_CO complex to the hydrogenase, nickel is transferred to the precursor with the help of HypA and HypB. The last step of catalytic core assembly involves proteolytic cleavage of the C-terminal tail of the large subunit after nickel insertion [19].

Disruption of *hypA* or *hypB* genes in various microorganisms resulted in hydrogenase deficiency that can be partially overcome by supplementation of nickel in the growing media [1], [2], [3], [4], [5], [6], [7]. HypA is a metallochaperone that binds both zinc and nickel. The zinc ion is bound with nanomolar affinity by two conserved CxxC motifs in HypA and it stabilizes the loop structure in HypA [20], [21], [22], [23]. HypA binds nickel via the conserved residues His-2 and Glu-3 [22], [23]. Mutagenesis study suggests that nickel binding via these two residues is essential for hydrogenase maturation [20]. HypB is a metal-binding GTPase. Mutagenesis study showed that both metal binding [24], [25] and GTP hydrolysis activity [3], [20], [26] are required for hydrogenase maturation.

How HypA and HypB facilitate nickel insertion into hydrogenase is unclear. Heterodimers of HypA/HypB from *Escherichia coli* and *Helicobacter pylori* were detected in cross-linking experiments and these complex formation were independent of nickel and GTP [20], [21]. Although HypA and HypB are known to be interacting partners, the significance of such interaction to hydrogenase maturation has never been addressed. *Archaeoglobus fulgidus* is a hyperthermophilic archeae that can grow organoheterotrophically, using a variety of carbon-based energy sources, and lithoautotrophically on hydrogen, thiosulphate and carbon dioxide. We have previously determined the crystal structure of HypB from *A. fulgidus* (AfHypB; PDB code: 2WSM). Based on scanning mutagenesis, we identified that the unstructured N-terminal region of the HypB GTPase domain is responsible for HypA binding. We further demonstrated that amino acid substitutions of HypB which disrupt HypA/HypB interaction also abolished *in vivo* hydrogenase maturation. These observations suggested that interaction between HypA and HypB is essential for nickel insertion to hydrogenase precursor to produce mature enzyme. Based on the proximity between the HypA and metal binding sites in HypB, possible role of HypA/HypB interaction for hydrogenase maturation is discussed.

## Results

### HypA and HypB from *A. fulgidus* form 1∶1 heterodimer in solution

**Figure 1:**
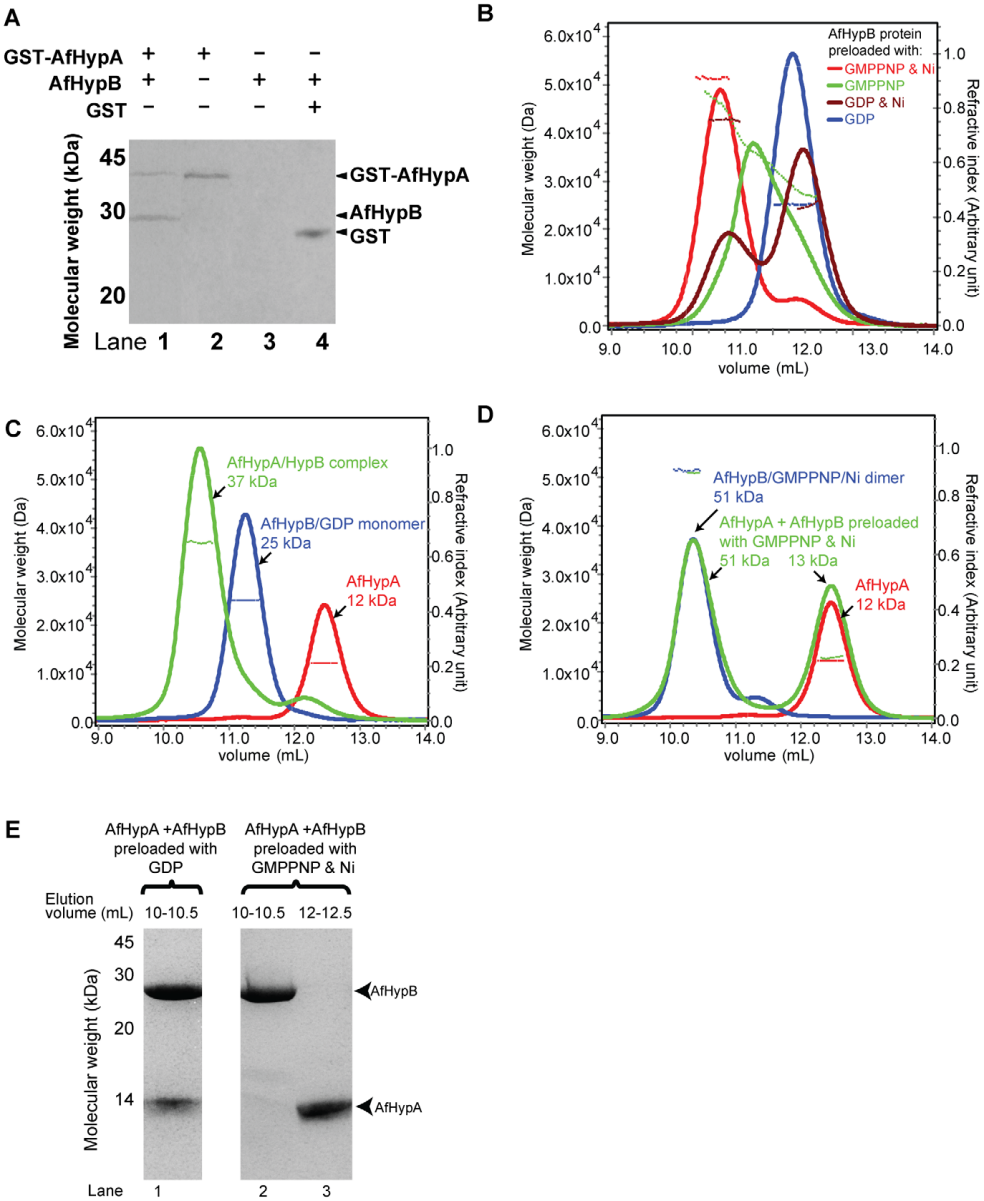
AfHypA and AfHypB form 1∶1 heterodimeric complex. (A) Cell lysate containing GST-tagged AfHypA were loaded on to glutathione resin with (lane 1) or without (lane 2) AfHypB. After extensive washing, GST-AfHypA and AfHypB were co-eluted with 20 mM glutathione (lane 1). In contrast, no co-elution was observed in the resin (lane 3) and the GST (lane 4) controls. (B) Purified AfHypB dimer preloaded with various ligands were subjected to SEC/LS analysis. Refractive index (continuous lines) and molecular weight (dotted lines) were plotted in the chromatogram. AfHypB preloaded with both GMPPNP and Ni (red) was eluted as a major peak with observed molecular weigh 51 kDa. AfHypB preloaded with GMPPNP (green) showed a broadened peak with observed molecular weight ∼36 kDa. AfHypB preloaded with Ni (brown) showed two eluted peaks with observed molecular weight 42 kDa and 25 kDa. AfHypB preloaded with GDP (blue) was eluted as a monomer with observed molecular weight of 25 kDa. (C) Mixture of equimolar of AfHypA and AfHypB preloaded with GDP (green) was analyzed by SEC/LS. AfHypA and AfHypB were eluted as a complex of 37 kDa. Individual elution profiles of AfHypA (red) and AfHypB preloaded with GDP (blue) were included for comparison. The corresponding observed molecular weight of each peak was labeled in the chromatogram. (D) Similarly, mixture of equimolar of AfHypA and AfHypB preloaded with GMPPNP and Ni (green) was analyzed by SEC/LS. AfHypA and AfHypB were eluted separately in two peaks of molecular weight 51 kDa and 13 kDa. Individual elution profiles of AfHypA (red) and AfHypB preloaded with GMPPNP and Ni (blue) were plotted for comparison. (E) The eluted peaks of interest were analyzed by SDS-PAGE. AfHypB preloaded with GDP were co-eluted with AfHypA (lane 1), in contrast, AfHypB dimer preloaded with GMPPNP and Ni was eluted separately (lane 2 and 3).

The interaction between *A. fulgidus* HypA (AfHypA) and HypB (AfHypB) was demonstrated by *in vitro* pull-down. AfHypB was mixed with cell lysate of *E. coli* overexpressing GST-tagged AfHypA and loaded onto the glutathione resin. After extensive washing with phosphate buffered saline, AfHypB was co-eluted with GST-AfHypA (lane 1, figure 1A). In contrast, no elution of AfHypB was detected in the resin and the GST controls (lane 3 and 4, figure 1A). The identity of AfHypB in the pull-down assay was confirmed by mass spectroscopy analysis of trypsinized fragments. The result suggests that AfHypA interacts with AfHypB.

### *A. fulgidus* HypB only forms stable dimer in the presence of both GMPPNP and nickel

Previous study showed that HypB forms dimer in the presence of GTP or Ni. Here, we measured the molecular weight of AfHypB preloaded with GMPPNP and/or Ni (Figure 1B) by size exclusion chromatography with light scattering (SEC/LS). AfHypB preloaded with GDP was eluted as a single peak with a molecular weight of 25 kDa, which is consistent with the theoretical molecular weight of monomeric AfHypB (24.8 kDa). AfHypB preloaded with Ni was eluted as two peaks with molecular weights of 42 kDa and 25 kDa, and that preloaded with GMPPNP only was eluted as a broadened peak with molecular weights of ∼36 kDa, indicating the presence of both monomeric and dimeric AfHypB in the samples (Figure 1B). In contrast, AfHypB preloaded with both GMPPNP and Ni was eluted as a single major peak with a molecular weight of 51 kDa, suggesting AfHypB forms a stable dimer in the presence of both ligands.

### *A. fulgidus* HypA form complex with HypB monomer preloaded with GDP but not with HypB homodimer preloaded with GMPPNP and Ni

Next, we addressed whether the oliogmeric state of HypB affects its interaction with HypA. AfHypA was mixed with AfHypB preloaded with GDP (monomeric form of AfHypB) or AfHypB preloaded with GMPPNP and Ni (dimeric form of AfHypB), and the molecular weights of the protein samples were measured by SEC/LS. When AfHypA was mixed with the monomeric form of AfHypB (preloaded with GDP), the two proteins was co-eluted at 10.5 ml (Figure 1E, lane 1) with a molecular weight of 37 kDa (Figure 1C), which is consistent with the theoretical molecular weight of 36.8 kDa for 1∶1 dimer of AfHypA-AfHypB. When AfHypA was mixed with the dimeric form of AfHypB (preloaded with GMPPNP and Ni), two elution peaks were observed. The first peak at 10.3 mL with an observed molecular weight of 51 kDa accounted for AfHypB dimer, which identity was confirmed by SDS-PAGE (Figure 1E, lane 2). The second peak at 12.5 mL with an observed molecular weight of 13 kDa accounts for AfHypA (Figure 1E, lane 3). These results suggest that AfHypA can only interact with the monomeric GDP-bound form of AfHypB, but cannot interact with the dimeric form of AfHypB.

### The unstructured N-terminal residues of *A. fulgidus* HypB are required for HypA/HypB interaction

**Figure 2:**
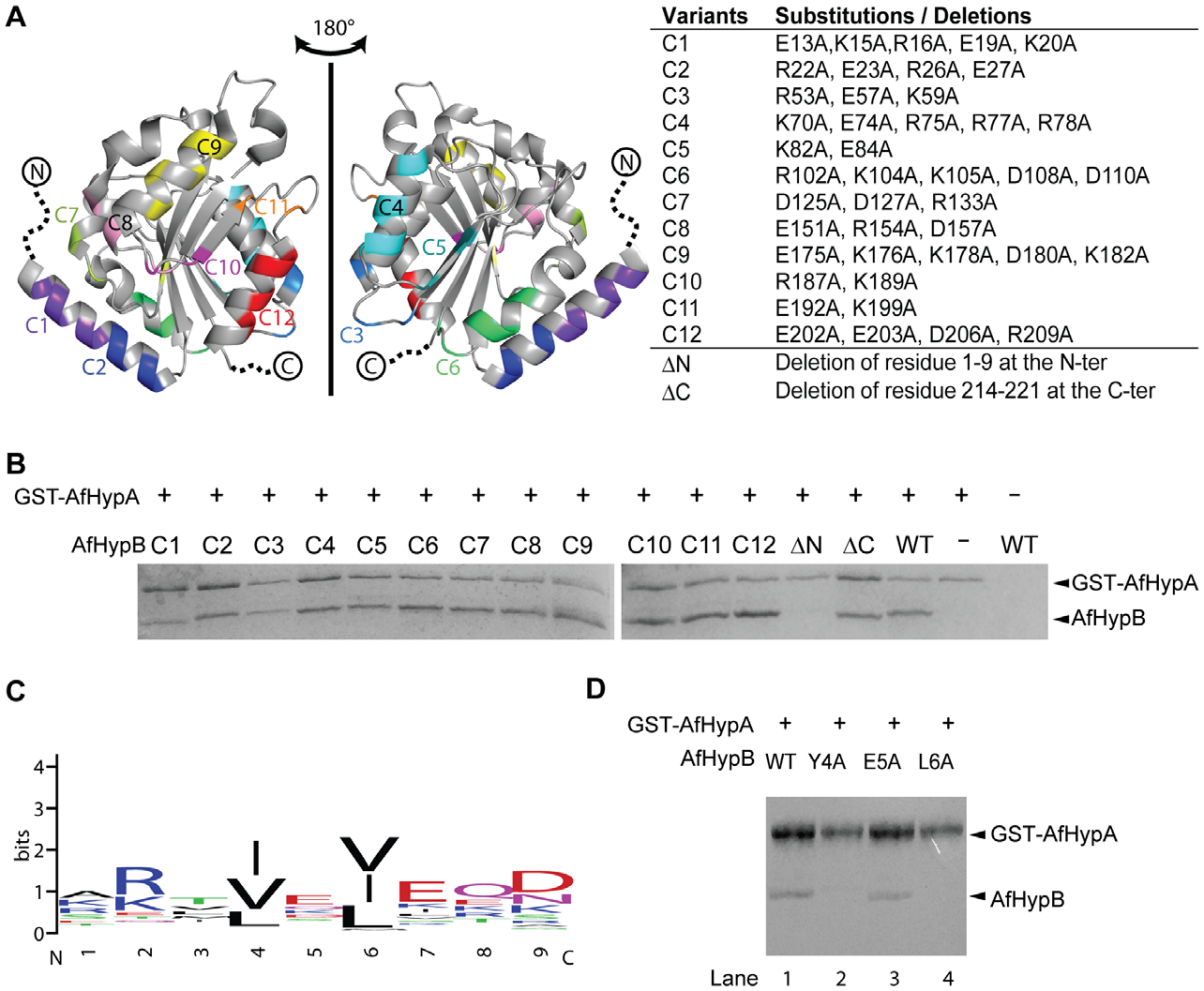
The unstructured N-terminal residues of AfHypB is required for HypA/HypB interaction. (A) Design of variants to map the HypA binding site on HypB. Twelve clustered-charge-to-alanine variants designated C1–C12, were created according to the close proximity of the charge residues in the crystal structure of AfHypB (PDB code: 2WSM). Residues 1–9 and 214–221 were unstructured in AfHypB. Two truncation variants, ΔN and ΔC, were created to investigate the role of these unstructured residues. (B) Interaction of AfHypB variants with AfHypA was tested by *in vitro* pull-down assay. Cell lysate containing GST-AfHypA and HypB variants was loaded on to glutathione-resin. Bound proteins were eluted by 20 mM glutathione after extensive washing. All the HypB surface charge mutants and C-terminal mutant were co-eluted with HypA suggesting that interaction was not disrupted by the mutations. N-terminal truncated HypB mutant was incapable to be co-eluted with HypA indicating that the first nine amino acid residues of AfHypB is required to interact with HypA. (D) Residues 4 and 6 (numbering according to the sequence of AfHypB) are conserved among HypB. The sequence logo representation was generated by the program WEBLOGO [35], [36] using 62 non-redundant (with <90% identity) sequences from the NCBI non-redundant database. Aliphatic hydrophobic residues are always found at these two positions. (E) Interaction of AfHypB variants Y4A, E5A and L6A with AfHypA was tested by *in vitro* pull-down assay. The variant E5A (lane 3) retained its interaction with AfHypA like the wild-type AfHypB (lane 1). Y4A (lane 2) and L6A (lane 4) variants were incapable to interact with AfHypA indicating that both residues are required to interact with HypA.

In an attempt to map the HypA binding site on HypB, we performed scanning mutagenesis on HypB. Based on the crystal structure of HypB from *A. fulgidus* (PDB: 2WSM), surface charged residues (Asp, Glu, Lys and Arg) on HypB were grouped into 12 clusters according to their proximity in its 3-dimentional structure. Twelve HypB variants were created by substituting residues in each cluster with alanine (figure 2A). In the crystal structure of AfHypB, residues 1–9 at the N-terminus and residues 215–221 at the C-terminus are disordered. This N-terminal region is also disordered in the crystal structures of *M. jannaschii* HypB [27]. To understand the role of these disordered residues, two additional truncation variants of HypB were created: an N-terminal truncated HypB variant (ΔN) with residues 1–9 deletion and a C-terminal truncated HypB variant (ΔC) with residues 215–221 deletion. To test the interaction between these AfHypB variants with AfHypA, *in vitro* pull-down assay using GST-AfHypA as bait was performed. All AfHypB variants were expressed as soluble proteins found in soluble fraction of crude lysate. Variants of AfHypB were mixed with GST-AfHypA, and loaded onto glutathione resin. After extensive washing, the bound proteins were eluted with 20 mM GSH and analyzed by 12.5% SDS-PAGE (figure 2B). The C1–C12 and the ΔC variants were co-eluted with GST-AfHypA, suggesting that these surface charged residues and the C-terminal unstructured residues are unnecessary for HypA/HypB interaction. On the other hand, the ΔN variant of HypB failed to co-elute with GST-HypA, showing that truncation of the unstructured N-terminal residues of HypB abolished interaction with HypA.

### Two conserved hydrophobic residues on *A. fulgidus* HypB are required to interact with HypA

We have shown that the truncation of residues 1–9 of AfHypB abolished the interaction between HypA and HypB. Sequence alignment suggests that there are two conserved hydrophobic residues, Tyr-4 and Leu-6, in this region (Figure 2C). To test if these two residues are involved in HypA interaction, two AfHypB variants, Y4A and L6A, were constructed by site-directed mutagenesis. An E5A variant was also constructed to test the role of the non-conserved residue Glu-5. All three variants were expressed as soluble proteins found in soluble fraction of crude cell lysate. *In vitro* pull-down assay was performed using GST-AfHypA as bait. Our results showed that wild-type AfHypB and the E5A variant were co-eluted with AfHypA (Figure 2D, lane 3 and 5), suggesting that substitution with alanine at Glu-5 did not affect the HypA and HypB interaction. On the other hand, GST-AfHypA did not pull down the Y4A and L6A variants of AfHypB (Figure 2D, lane 4 and 6), suggesting that the two conserved non-polar residues, Tyr-4 and Leu-6, of AfHypB are required for interaction with HypA.

### Two conserved hydrophobic residues on *E. coli* HypB are required to interact with HypA

**Figure 3:**
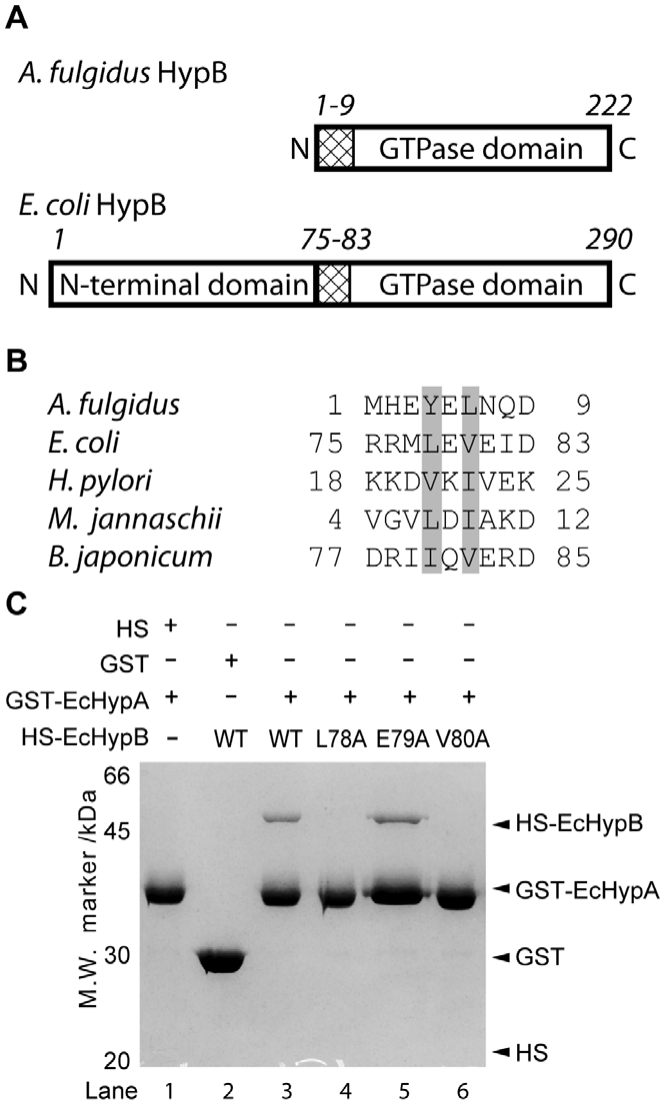
Two conserved hydrophobic residues located at the N-terminus of the GTPase domain of HypB are required for HypA/HypB interaction. (A) Both *A. fulgidus* HypB (AfHypB) and *E. coli* HypB (EcHypB) have a GTPase domain. The unstructured N-terminal residues of AfHypB (residues 1–9) correspond to residues 75–83 of EcHypB located at the N-terminus of the GTPase domain. (B) Sequence alignment of HypB from five selected species was shown. The two conserved hydrophobic residues were shaded. (C) Interaction between variants of EcHypB and EcHypA were tested by GST pull-down assay. EcHypB was expressed with an N-terminal His-SUMO (HS) tag. Cell lysate expressing HS-EcHypB or its variants was mixed with that expressing GST-EcHypA, and was loaded onto glutathione-resin. After extensive washing, wild-type HS-EcHypB and GST-EcHypA was co-eluted with 20 mM GSH (lane 3). While E79A variant of EcHypB retained its interaction with HypA to be co-eluted with GST-EcHypA (lane 5), no co-elution was observed for the L78A and V80A variants (lane 4 and 6). No co-elution was observed for the negative control experiments (lane 1 and 2), suggesting the interaction between EcHypA and EcHypB is specific.

*E. coli* HypB (EcHypB) has an extra N-terminal domain in addition to the GTPase domain comparing with AfHypB. Residues 1–9 of AfHypB correspond to residues 75–83 in *E. coli* HypB (EcHypB), which is the N-terminus of the GTPase domain of HypB (Figure 3A). The conserved non-polar residues, Tyr-4 and Leu-6, of AfHypB correspond to Leu-78 and Val-80 of EcHypB (Figure 3B). To test if these residues are involved in HypA/HypB interaction, EcHypB variants, L78A, E79A and V80A, were constructed by site-directed mutagenesis. These variants of EcHypB were expressed with an N-terminal His-SUMO (HS) tag and their interaction with EcHypA was tested by *in vitro* GST-pull-down assay (Figure 3C). EcHypB and its variants were expressed as soluble proteins found in the soluble fraction of crude lysate. Lysate containing HS-EcHypB variants and the lysate containing GST-EcHypA were mixed and loaded on to GSH-resin for pull-down assay. Our results showed that wild-type HS-EcHypB was co-eluted with GST-EcHypA (Figure 3C, lane 3). The identity of HS-EcHypB was confirmed by mass spectroscopy analysis of trypsinized fragments. Like wild-type EcHypB, the E79A variant retained its interaction with GST-EcHypA. On the other hand, GST-EcHypA did not pull down the L79A and V80A variants of EcHypB (Figure 3C, lane 4 and 6), suggesting that the two conserved hydrophobic residues, Leu-78 and Val-80, of EcHypB are required for interaction with HypA. This observation is consistent with the observation in the pull down experiment of AfHypA and AfHypB.

### HypA/HypB interaction is required for hydrogenase maturation in *E. coli*

**Figure 4:**
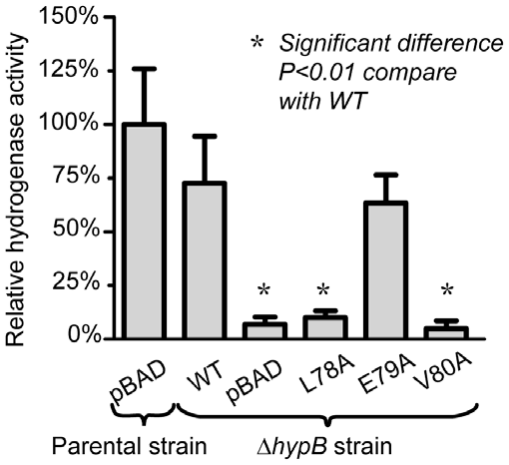
Mutations that disrupted HypA/HypB interactions also abolished restoration of hydrogenase activity *in vivo*. The Δ*hypB* strain was transformed with the empty vector control (pBAD), wild-type pBAD-EcHypB and its mutants (L78A, E79A or V80A). The hydrogenase activity of the cell lysate was compared to that of the parental strain. Expression of wild-type HypB recovered 73±21% hydrogenase activity. Noteworthy, for the mutations (L78A and V80A) that disrupted HypA/HypB interactions, the hydrogenase activity was significantly reduced, and was similar to that for the empty vector control. The p-values were obtained by Dunnett's Multiple Comparison Test to compare each setup with Δ*hypB* strain-WT.

To test if HypA/HypB interaction is required for its biological function, *in vivo* hydrogenase reactivation experiment was carried out. Hydrogenase activity of *E. coli* Δ*hypB* strain (F-, *Δ(araD-araB)567*, *ΔlacZ4787*(::rrnB-3), *λ^−^*, *ΔhypB731::kan*, *rph-1*, *Δ(rhaD-rhaB)568*, *hsdR514*) expressing HypB and HypB variants were compared to the activity of the parental strain (F-, *Δ(araD-araB)567*, *ΔlacZ4787*(::rrnB-3), *λ^−^*, *rph-1*, *Δ(rhaD-rhaB)568*, *hsdR514*) (Figure 4).

Δ*hypB* strain with transformed pBAD-EcHypB showed a relative activity of 73±21% compared to the parental strain, suggesting that HypB can complement the loss of HypB gene. Transformation of the E79A mutant of EcHypB showed a relative activity of 63±13%, suggesting that the mutation of E79A did not affect the hydrogenase maturation significantly. On the other hand, transformation of the L78A and V80A mutants resulted in only 10±3% and 5±4% relative activity, respectively. These values were comparable to 7±3% for the empty vector, suggesting that the mutations of L78A and V80A abolished *in vivo* hydrogenase maturation. Our results suggest that the L78A and V80A mutations disrupted HypA and HypB interaction and abolished *in vivo* maturation of hydrogenase in *E. coli*.

## Discussion

In this study, we addressed the question of how HypB interacts with HypA, and if such interaction plays a role in hydrogenase maturation. We demonstrated that *A. fulgidus* HypA form a 1∶1 heterodimer with the monomeric GDP-bound form of HypB, but does not interact with the dimeric form of HypB preloaded with GMPPNP and Ni. We showed that residues located at the N-terminus of the GTPase domain of HypB are required for interaction with HypA. Single residue substitution of two conserved hydrophobic residues (Y4 and L6 in *A. fulgidus* HypB, L78 and V80 in *E. coli* HypB) in this region disrupted HypA/HypB interaction and abolished hydrogenase maturation in *E. coli*. We have extended this finding to *Helicobacter pylori* HypA/HypB interaction, and showed that the substitutions of N-terminal residues of *H. pylori* HypB also break the interaction with HypA [28]. Taken together, our results showed that HypA/HypB interaction is required for hydrogenase maturation.

**Figure 5:**
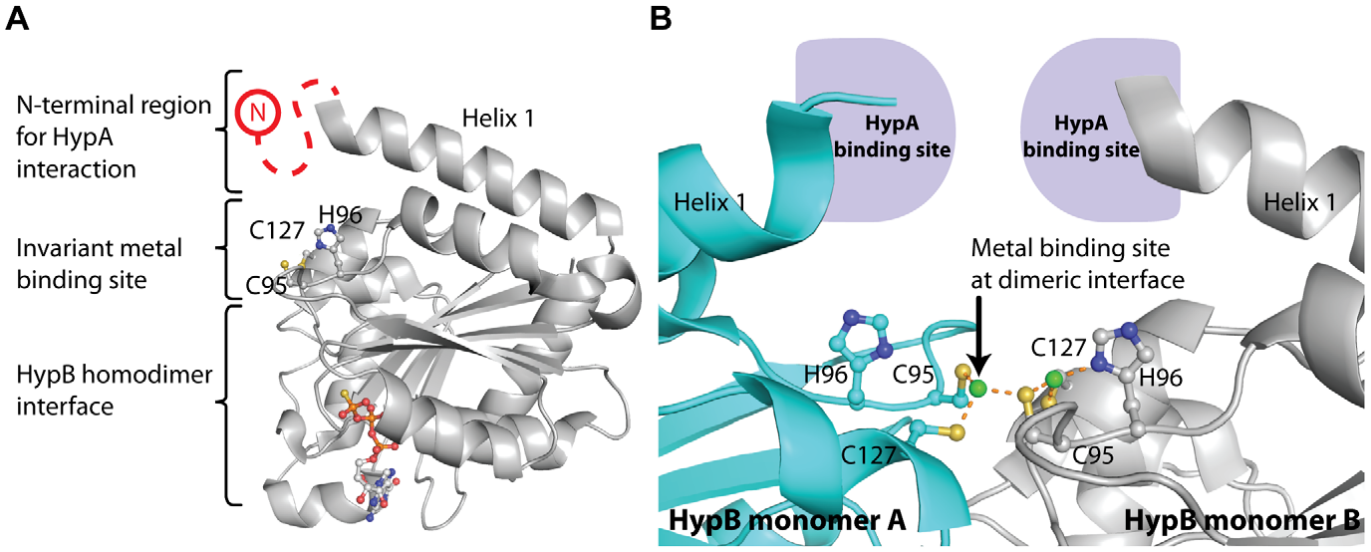
Close proximity of the HypA and metal binding sites of HypB suggests possible metal transfer between HypA and HypB. (A) The N-terminal residues of HypB, responsible for HypA binding, were not resolved in the crystal structures of HypB (red dashed line). HypB contains an invariant metal binding site composed of two cysteine residues and one histidine residue (C95, H96 and C127 in *M. jannaschii* HypB, PDB code: 2HF8). The close proximity between the metal binding site at the dimeric interface and the HypA binding site at the N-terminus of the GTPase domain suggest that HypA/HypB interaction may facilitate transfer of nickel between the two proteins. (B) Binding of HypA to HypB may facilitate transfer of nickel between HypA and the metal binding sites (C95, H96 and C127 in *M. jannaschii* HypB) situated at the dimeric interface of HypB. This metal binding site at the dimeric interface was occupied by zinc (green). Dimerization of HypB may induce steric hinderance between the HypA binding sites and prevent HypA from interacting with the dimeric form of HypB.

How HypA/HypB interaction contributes to hydrogenase maturation is poorly understood. Previous studies showed that HypA and HypB are responsible for the delivery of nickel ion to the active site of [NiFe] hydrogenases [1], [2], [3], [4], [5], [6], [7]. It has been demonstrated that binding of GTP induces HypB dimerization [20], [27], [29]. As observed in the crystal structure of *Methanococcus jannaschii* HypB in complex with GTPγS, metal ions are chelated by three invariant residues Cys-95, His-96 and Cys-127 [27]. Mutagenesis studies have suggested that these invariant residues are essential to hydrogenase maturation [24], [25]. Gasper *et al.* observed that GTP-dependent dimerization brought Cys-95 and Cys-96 from each chain of HypB together to allow both chains to bind a nickel ion at the interface (Figure 5A) [27]. GTP hydrolysis was shown to be essential for the maturation of hydrogenase [3], [20], [26]. It is possible that GTP hydrolysis will dissociate the HypB dimer, and release the nickel ion bound at the dimeric interface to the hydrogenase. This hypothesis is supported by the observation that mutations disrupting GTP-dependent dimerization of HypB also resulted in reduced hydrogenase activity [30], [31]. In this study, we showed that the N-terminal residues of the GTPase domain of HypB is responsible for binding HypA. It is noteworthy that the N-terminus of the GTPase domain is located in close proximity to the invariant residues responsible for metal binding (Figure 5B). The close proximity of the HypA interaction site and the metal binding site of HypB may facilitate transfer of nickel ions between HypA and HypB. Here we suggest that the nickel ion is transferred from HypA to HypB via the formation of HypA/HypB complex, and is then delivered to the catalytic core of hydrogenase upon GTP hydrolysis.

Another point of interest is when HypB forms dimer, N-terminus of helix 1 on each copy of HypB is pointing towards each other. We anticipate that dimerization of HypB may induce steric hinderance between the HypA binding sites and prevent HypA from interacting with the dimeric form of HypB (Figure 5B). Taken together, HypA may function as a metal chaperone by binding to HypB. After loading of nickel and binding of GTP to HypB, HypB dimerizes and HypA dissociates from HypB. HypB dimer in complex with GTP and Ni then delivers its bound nickel to hydrogenase precursor upon GTP hydrolysis.

## Materials and Methods

### Cloning and mutagenesis

Full length *hypA* and *hypB* genes were amplified by PCR from the *A. fulgidus* (ATCC: 49558) and *E. coli* (strain DH5α) genomic DNA, and cloned into the expression vector pRSET-His-SUMO (an in-house expression plasmid based on pRSET-A (Invitrogen) with an N-terminal His-SUMO tag), pRSET-GST (an in-house expression plasmid based on pRSET-A (Invitrogen) with an N-terminal GST fusion tag) and pBAD-A (Invitrogen). All mutants of HypB were constructed by QuikChange mutagenesis kit from Stratagene on constructs pRSET-His-SUMO-AfHypB, pRSET-His-SUMO-EcHypB and pBAD-EcHypB. Constructs created and the primers used are listed in table S1. All constructs were verified by DNA sequencing.

### Protein Expression

All proteins were expressed by transforming the corresponding expression plasmids into *E. coli* BL21 (DE3) pLysS host. The bacterial cells were grown in LB medium at 37°C supplemented with 100 µg/mL ampicillin and 50 µg/mL chloramphenicol. Protein expression was induced by addition of 0.4 mM isopropyl-D-1-thiogalactopyranoside when OD600 reached 0.8. Cells were allowed to grow overnight at 25°C and then were harvested by centrifugation at 5,000× g, 15 min, 4°C. Cell pellets were stored in −20°C freezer before use.

### Purification of AfHypA and AfHypB

Cell pellet expressing His-SUMO-AfHypA or His-SUMO-AfHypB from 1 L culture was resuspended in 20 mL of lysis buffer (20 mM Tris-HCl pH 7.5 containing 500 mM NaCl, 50 mM imidazole and 1 mM TCEP), and lysed by sonication. Cell debris was removed by centrifugation at 13000× g for 30 min, and the supernatant was loaded onto a 5 mL HiTrap™ IMAC Column (GE Healthcare) charged with Ni (II) ion. Column was pre-equilibrated with the lysis buffer. After extensive washing with the lysis buffer, His-tagged protein was eluted with 300 mM imidazole in lysis buffer. Eluent was supplemented with 0.5 mg of His-tagged SUMO protease per 1 L of culture and dialyzed in 20 mM Tris-HCl pH 7.5 containing 200 mM NaCl and 1 mM TCEP at 4°C overnight. After proteolytic cleavage, the His-SUMO tag was removed by nickel affinity chromatography. The column was washed with 20 mM Tris-HCl pH 7.5, 200 mM NaCl, 50 mM imidazole and 1 mM TCEP. Desired tag-free HypA or HypB was found in the flow-through and washing fractions. It was concentrated to a concentration of 10 mg/mL, and loaded onto a HiLoad 26/60 Superdex 75 column (GE Healthcare) equilibrated with 50 mM Tris-HCl buffer pH 7.5, 0.2 M NaCl and 1 mM TCEP. Eluted fractions were analyzed by SDS-PAGE. Purified HypA was eluted at elution volume of ∼205 ml and purified HypB was eluted at elution volume of ∼165 ml. Apo-AfHypB was prepared by extensive dialysis in 50 mM Tris-HCl buffer pH 7.5, 0.2 M NaCl, 1 mM TCEP and 5 mM EDTA to remove the bound metal and nucleotide [31]. EDTA was later removed by dialysis again. Purified proteins were snap frozen with liquid nitrogen, and stored in −80°C deep freezer before use.

### Determination of molecular weight of AfHypB preloaded with GDP, GMPPNP with and without nickel by size-exclusion-chromatograpghy/light-scattering (SEC/LS)

AfHypB (0.5 mM) was mixed with 5 mM GMPPNP or GDP, with or without 1 mM NiCl_2_. After removal of excess ligands by gel filtration, the AfHypB samples were loaded to a Superdex-75 HR10/30 column (GE Healthcare) equilibrated with 50 mM Tris pH 7.5, 0.2 M NaCl, 5 mM MgCl_2_ and 1 mM TCEP. The molecular weight of the AfHypB samples was measured by a downstream multiple-angle laser light scattering detector (Mini-DAWN light scattering detector, Wyatt Technology). Data were analyzed using Astra version 5.3.4.18 (Wyatt Technology), following the manufacturer's instructions.

### Determination of molecular weight of AfHypA/AfHypB complex

The molecular weight of *A. fulgidus* HypA/HypB complex was determined using SEC/LS. Protein samples were analyzed by loading onto a Superdex-75 HR10/30 column (GE Healthcare) equilibrated with the 50 mM Tris pH 7.5, 0.2 M NaCl and 1 mM TCEP, and eluted at room temperature at a flow rate of 0.5 mL/min. The column was connected downstream to a multiple-angle laser light scattering (Mini-DAWN light scattering detector, Wyatt Technology). Data were analyzed using Astra version 5.3.4.18 (Wyatt Technology), following the manufacturer's instructions.

### GST pull-down assay

Interaction between AfHypA and AfHypB was tested by GST pull-down assay using GST-AfHypA as a bait. 50 mL overnight culture of *E. coli* cells expressing GST-AfHypA and His-SUMO-AfHypB were collected by centrifugation and lysed by sonication in 5 mL phosphate buffered saline (PBS). To remove the His-SUMO tag, 0.5 mg of SUMO protease was added to the clear cell lysate of His-SUMO-AfHypB. After incubation at 25°C for 1 hour, the lysates containing AfHypB and GST-AfHypA were mixed together and loaded onto 0.1 mL glutathione(GSH)-resin. After extensive washing PBS, bound proteins were eluted with 20 mM Tris pH 8.0, 0.1 M NaCl and 20 mM reduced GSH. The eluted proteins were analyzed by a 15% SDS-PAGE stained with Coomassie blue.

The GST pull-down assay for EcHypA and EcHypB was performed as described for AfHypA and AfHypB with the exception that the His-SUMO tag of EcHypB was not cleaved so that the bands of GST-EcHypA and His-SUMO-HypB can be clearly distinguished in SDS-PAGE analysis

### Hydrogenase reactivation assay

Hydrogenase reactivation from crude cell extract was measured by a modified method based on the procedures of Ballantine and Boxer [32] and Zhang *et al.* [33]. The *E. coli* Δ*hypB* strain (*F^−^, Δ(araD-araB)567, ΔlacZ4787 (::rrnB-3), λ^−^, ΔhypB731::kan, rph-1, Δ(rhaD-rhaB)568, hsdR514*) (Keio collection no.: JW2697-1), obtained from the Keio collection, *E. coli* Genetic Stock Center [34], was transformed with the wild-type pBAD-EcHypB or its mutants. The Δ*hypB* genotype and the pBAD-EcHypB plasmid were selected by 50 µg/ml kanamycin and 100 µg/ml ampicillin, respectively. Starter culture was grown from single colonies in LB medium at 37°C. After 2% (v/v) inoculation, the *E. coli* cells were grown anaerobically using 50 mL sealed plastic tubes in buffered TGYEP medium (10 g/L tryptone, 5 g/L yeast extract, 0.8% glycerol, 69 mM K_2_HPO_4_, and 22 mM KH_2_PO_4_) supplemented with 15 mM sodium fumarate, 1 µM sodium molybdate, and 1 µM sodium selenite for 16 hours. The expression was induced by adding 1 µM arabinose. Cells were harvested by centrifugation and washed with 50 mM potassium phosphate buffer, pH 7.0, and resuspended in the same buffer containing 1 mM dithiothreitol and 0.2 mM phenylmethylsulfonyl fluoride. Crude cell extracts were prepared by sonication and subsequent centrifugation at 13,000× g for 30 min at 4°C. The protein concentrations of crude cell extracts were determined by the Bradford protein assay kit (BioRad) using bovine serum albumin as standard.

Total hydrogenase activity of the crude cell extracts was measured by hydrogen-dependent reduction of benzyl viologen. Reactions were prepared in a septum-sealed cuvette. Sealed cuvette containing 2.5 mL of 50 mM potassium phosphate, pH 7.0 was degassed thoroughly by vacuum pump followed by bubbling with 95% N_2_ and 5% H_2_ for 15 minutes. 200 µL solution of 0.05% (w/v) sodium dithiolate was injected into the cuvette to remove the remaining oxidative species. To start a reaction, 300 µL of degassed diluted crude cell extract (at 0.2 mg/mL of total protein) was injected into the cuvette. The initial OD600 should be in the range of 0.2–0.5 to ensure there was no remaining oxidative species in the reaction. OD600 was monitored at 25°C for 15 minutes. The amount of benzyl viologen reduced was quantified by changes in OD600 using the extinction coefficient of 7400 M^−1^ cm^−1^. Hydrogenase activity was measured as nanomol of benzyl viologen reduced per min per mg of total protein. Activity measurement of each condition was repeated for three times in individual experiment. Hydrogenase activity of the wild type parental strain *E. coli* (*F-, Δ(araD-araB)567, ΔlacZ4787(::rrnB-3), λ^−^, rph-1, Δ(rhaD-rhaB)568, hsdR514*) transformed with pBAD-A was measured along with each batch of reaction as positive control and relative standard.

## Supporting Information

Table S1 **Constructs and Primers used.** Desired DNA fragment was amplified by PCR the from genomic DNA using the corresponding primer pair, followed by restriction digestion to create compatible ends on both inserts and plasmids. DNA ligation was performed to generate the desired plasmid. Restriction enzyme recognition sites on the primers listed are in lower case letters. Quikchange mutagenesis was performed using the corresponding primers to generate the variant construct from the parental wild-type construct. Mutation sites that are not complementary to the parental construct on the primers are in lower case letters.
(DOC)
