# Supplementary material for: Interaction between Hydrogenase Maturation Factors HypA and HypB Is Required for [NiFe]-Hydrogenase Maturation
Source: PLoS One. 2012 Feb 27;7(2):e32592. doi: 10.1371/journal.pone.0032592 (PMC3287977; doi:10.1371/journal.pone.0032592)
Supplement: Table S1 — Constructs and Primers used. Desired DNA fragment was amplified by PCR the from genomic DNA using the corresponding primer pair, followed by restriction digestion to create compatible ends on both inserts and plasmids. DNA ligation was performed to generate the desired plasmid. Restriction enzyme recognition sites on the primers listed are in lower case letters. Quikchange mutagenesis was performed using the corresponding primers to generate the variant construct from the parental wild-type construct. Mutation sites that are not complementary to the parental construct on the primers are in lower case letters. (DOC) [file pone.0032592.s001.doc]

Table S1 Constructs and Primers used.

| **Constructs** | **Description** | **Primers used (5' to 3')** | **Method to generate constructs** |
| --- | --- | --- | --- |
| **Plasmid used for construction of fusion tag** | | |  |
| pRSET-A | Commercially available expression vector obtained from Invitrogen | - |  |
| pBAD-A | Commercially available expression vector obtained from Invitrogen | - |  |
| **Recombinant AfHypA expression vectors** | | | |
| pRSET-GST-AfHypA | Expression vector for GST-tagged *A. fulgidus* HypA | TATGCAggatccATGCATGAAATGAGCTTTGC  TATGCAgaattcCTACACCTCCACAACTACG | Ligation of DNA fragments |
| pRSET-HS-AfHypA | Expression vector for His-SUMO-tagged *A. fulgidus* HypA | TATGCAaccggtGGAATGCATGAAATGAGCTTTGC  TATGCAgaattcCTACACCTCCACAACTACG | Ligation of DNA fragments |
| **Recombinant GST-EcHypA expression vector** | | | |
| pRSET-GST-EcHypA | Expression vector for GST-tagged *E. coli* HypA | TATGCAggatccATGCACGAAATAACCC  TATGCAgaattcTCACTCCTGGTCTATTTC | Ligation of DNA fragments |
| **Recombinant His-SUMO AfHypB and variants expression vectors** | | | |
| pRSET-HS-AfHypB | Expression vector for His-SUMO-tagged *A. fulgidus* HypB | TATGCAggatccATGCACGAGTATGAACTTAATC  TATGCAgaattcTCAATTCTGCCCGGAATC | PCR,restriction, ligation. |
| pRSET-HS-AfHypB-C1 | Alanine substitution at residues 13, 15,16,19,20 | | CAGGATTTGCTTGCCGCAAACgcagcaCTGGCAGcagcGAACAGAGAAGCGCTC | | --- | | GAGCGCTTCTCTGTTCgctgCTGCCAGtgctgcGTTTGCGGCAAGCAAATCCTG | | Quikchange mutagenesis |
| pRSET-HS-AfHypB-C2 | Alanine substitution at residues 22,23,26,27 | | GCTGGCAGAGAAGAACgcAgcAGCGCTCgcAGcGAGCGGGACTGTGGC | | --- | | GCCACAGTCCCGCTCgCTgcGAGCGCTgcTgcGTTCTTCTCTGCCAGC | | Quikchange mutagenesis |
| pRSET-HS-AfHypB-C3 | Alanine substitution at residues 53,57,59 | | CATTGAGAGAACAATCGAGgcaATCGGCAATgcaGTCgcaATTGGCGCCATGCTGG | | --- | | CCAGCATGGCGCCAATtgcGACtgcATTGCCGATtgcCTCGATTGTTCTCTCAATG | | Quikchange mutagenesis |
| pRSET-HS-AfHypB-C4 | Alanine substitution at residues 82, 84 | | GGAGATGTTGTCTCCgcaGCTGACTATgcagcaGTGgcagcaTTCGGAATTAAGGC | | --- | | GCCTTAATTCCGAAtgctgcCACtgctgcATAGTCAGCtgcGGAGACAACATCTCC | | Quikchange mutagenesis |
| pRSET-HS-AfHypB-C5 | Alanine substitution at residues 102,104,105,108,110 | | GAGAAGATTCGGAATTgcaGCAgcgGCGATAAGCACGGG | | --- | | CCCGTGCTTATCGCcgcTGCtgcAATTCCGAATCTTCTC | | Quikchange mutagenesis |
| pRSET-HS-AfHypB-C6 | Alanine substitution at residues 125,127,133 | | GCACATGATTTACCACgcaCTGgcagcaTTCTCCGCTTGTgctTTGCTCCTAATAGAG | | --- | | CTCTATTAGGAGCAAagcACAAGCGGAGAAtgctgcCAGtgcGTGGTAAATCATGTGC | | Quikchange mutagenesis |
| pRSET-HS-AfHypB-C7 | Alanine substitution at residues 151,154,157 | | CAATTTAATCTGCCCAGTGgcaTTTgctCTGGGAGAGAACTACgcgGTGGTGATGGTCAG | | --- | | CTGACCATCACCACcgcGTAGTTCTCTCCCAGagcAAAtgcCACTGGGCAGATTAAATTG | | Quikchange mutagenesis |
| pRSET-HS-AfHypB-C8 | Alanine substitution at residues 175, 176, 178, 180, 182 | | GGTTGAAAAGCATCCAgcaATTTTCgcaGTTGCAgctTTGATTGTAATCAAC | | --- | | GTTGATTACAATCAAagcTGCAACtgcGAAAATtgcTGGATGCTTTTCAACC | | Quikchange mutagenesis |
| pRSET-HS-AfHypB-C9 | Alanine substitution at residues 187, 189 | | GTTGGGGCAGATGTTgcagcaATGgcaGCAgctGCAgcgCTTATCAACCCGAG | | --- | | CTCGGGTTGATAAGcgcTGCagcTGCtgcCATtgctgcAACATCTGCCCCAAC | | Quikchange mutagenesis |
| pRSET-HS-AfHypB-C10 | Alanine substitution at residues 192, 199 | | CGAAGCTTATCAACCCGgcaGCTgcaATCATCGAGATGGACC | | --- | | GGTCCATCTCGATGATtgcAGCtgcCGGGTTGATAAGCTTCG | | Quikchange mutagenesis |
| pRSET-HS-AfHypB-C11 | Alanine substitution at residues | | GAGAGCTAAAATCATCgcgATGGACCTCAAAACCGGTgcaGGATTTGAGGAGTG | | --- | | CACTCCTCAAATCCtgcACCGGTTTTGAGGTCCATcgcGATGATTTTAGCTCTC | | Quikchange mutagenesis |
| pRSET-HS-AfHypB-C12 | Alanine substitution at residues 202,203, 206, 209 | | CCGGTAAAGGATTTgcagcaTGGATTgcaTTTTTGgcgGGGATTCTGAATGTG | | --- | | CACATTCAGAATCCCcgcCAAAAAtgcAATCCAtgctgcAAATCCTTTACCGG | | Quikchange mutagenesis |
| pRSET-HS-AfHypB-ΔN | AfHypB(residues 8-221) | | GAACAAACCGGTGGATCCTTGCTTGCCGAAAACAAG | | --- | | CTTGTTTTCGGCAAGCAAGGATCCACCGGTTTGTTC | | Quikchange mutagenesis |
| pRSET-HS-AfHypB-ΔC | AfHypB(residues 1-213) | | GAGGGGGATTCTGAATtgaCATAGCGATTCCGGG | | --- | | CCCGGAATCGCTATGtcaATTCAGAATCCCCCTC | | Quikchange mutagenesis |
| **Recombinant His-SUMO EcHypB and variants expression vectors** | | | |
| pRSET-HS-EcHypB | Expression vector for His-SUMO-tagged *E. coli* HypB | TATGCAggatccATGGGTATGTGTACAACATGCGGTTG  TATGCAgaattcCTATGCACATCGCTGTGTC | Ligation of DNA fragments |
| pRSET-HS-EcHypB-L78A | *E. coli* HypB mutant L78A | ATGAGCCAGCGTCGGATGgcGGAAGTCGAAATTGAC  GTCAATTTCGACTTCCgcCATCCGACGCTGGCTCAT | Quikchange mutagenesis |
| pRSET-HS-EcHypB-E79A | *E. coli* HypB mutant E79A | | CAGCGTCGGATGCTGGcgGTCGAAATTGACGTG | | --- | | CACGTCAATTTCGACcgCCAGCATCCGACGCTG | | Quikchange mutagenesis |
| pRSET-HS-EcHypB-V80A | *E. coli* HypB mutant V80A | | CGTCGGATGCTGGAAGcgGAAATTGACGTGCTG | | --- | | CAGCACGTCAATTTCcgCTTCCAGCATCCGACG | | Quikchange mutagenesis |
| **Recombinant EcHypB expression vectors for hydrogenase assay** | | | |
| pBAD-EcHypB | Expression vector for *E. coli* HypB | TATGCAccatggGTATGTGTACAACATGCGGTTG  TATGCAgaattcCTATGCACATCGCTGTGTC | Ligation of DNA fragments |
| pBAD-EcHypB-L78A | *E. coli* HypB mutant L78A | ATGAGCCAGCGTCGGATGgcGGAAGTCGAAATTGAC  GTCAATTTCGACTTCCgcCATCCGACGCTGGCTCAT | Quikchange mutagenesis |
| pBAD-EcHypB-E79A | *E. coli* HypB mutant E79A | | CAGCGTCGGATGCTGGcgGTCGAAATTGACGTG | | --- | | CACGTCAATTTCGACcgCCAGCATCCGACGCTG | | Quikchange mutagenesis |
| pBAD-EcHypB-V80A | *E. coli* HypB mutant V80A | | CGTCGGATGCTGGAAGcgGAAATTGACGTGCTG | | --- | | CAGCACGTCAATTTCcgCTTCCAGCATCCGACG | | Quikchange mutagenesis |
